# Education, intelligence, and amyotrophic lateral sclerosis: A Mendelian randomization study

**DOI:** 10.1002/acn3.51156

**Published:** 2020-08-18

**Authors:** Linjing Zhang, Lu Tang, Kailin Xia, Tao Huang, Dongsheng Fan

**Affiliations:** ^1^ Department of Neurology Peking University Third Hospital Beijing China; ^2^ Department of Epidemiology and Biostatistics School of Public Health Peking University Beijing China; ^3^ Key Laboratory of Molecular Cardiovascular Sciences (Peking University) Ministry of Education Beijing China; ^4^ Key Laboratory for Neuroscience National Health Commission/Ministry of Education Peking University Beijing China

## Abstract

**Objective:**

To systematically investigate causal relationships between educational attainment, cognitive‐related phenotypes, and amyotrophic lateral sclerosis (ALS).

**Methods:**

Summary statistics from genome‐wide association studies for educational attainment, math ability, highest math class taken, cognitive performance, intelligence, and ALS were used. A two‐sample Mendelian randomization (MR) design was applied to explore the potential causal associations between them.

**Results:**

Genetically predisposition to longer educational attainment and harder math class taken were associated with significantly lower statistical odds ratio of ALS. For per year increase in education completed there was a 21% lower (95% confidence interval [CI] = 27–14%) in risk for ALS. For per 1‐SD increase in the highest math class taken obtained there was a 19% lower (95% CI = 9–28%) in risk for ALS. Genetically predisposition to math ability, cognitive performance, and intelligence did not decrease the risk of ALS.

**Interpretation:**

This study provides genetic support for a causal association between higher educational attainment and a lower risk of ALS. The genes related to these phenotypes are involved in almost all aspects of neuron‐to‐neuron communication. ALS patients are occasionally accompanied by varying degrees of cognitive impairment. People with greater cognitive reserve may be better able to offset damages of degenerative brain changes associated with dementia or other brain diseases, such as Alzheimer's disease, Parkinson's disease, multiple sclerosis, or ALS.

## Introduction

Evidence from three case–control studies demonstrates that a longer duration of education is associated with a reduced incidence of amyotrophic lateral sclerosis (ALS).^1^, ^2^, ^3^ Meta‐analyses have shown that individuals with lower education are more likely to develop ALS than those with higher education completed, and the pooled odds ratio was 2.04 (95% CI: 1.58–2.62).^4^ However, the extent of individuals' educational attainment was strongly influenced by their socioeconomic status,^5^ behavior problems,^6^ physical health,^7^ and mental health,^7^ and observational studies are prone to suffer from confounding and other potential biases; randomized controlled trials cannot be performed due to ethical issues. Thus, whether such an association reflects causality remains to be further explored. Here, a two‐sample Mendelian randomization (MR) was performed to examine the causal effects of educational attainment and intelligence on the ALS risk. MR is a potentially robust method that can support the translation of observational correlations into causal relationships while minimizing biases due to confounding and reverse causation.^8^, ^9^, ^10^, ^11^

## Methods

**Figure 1:**
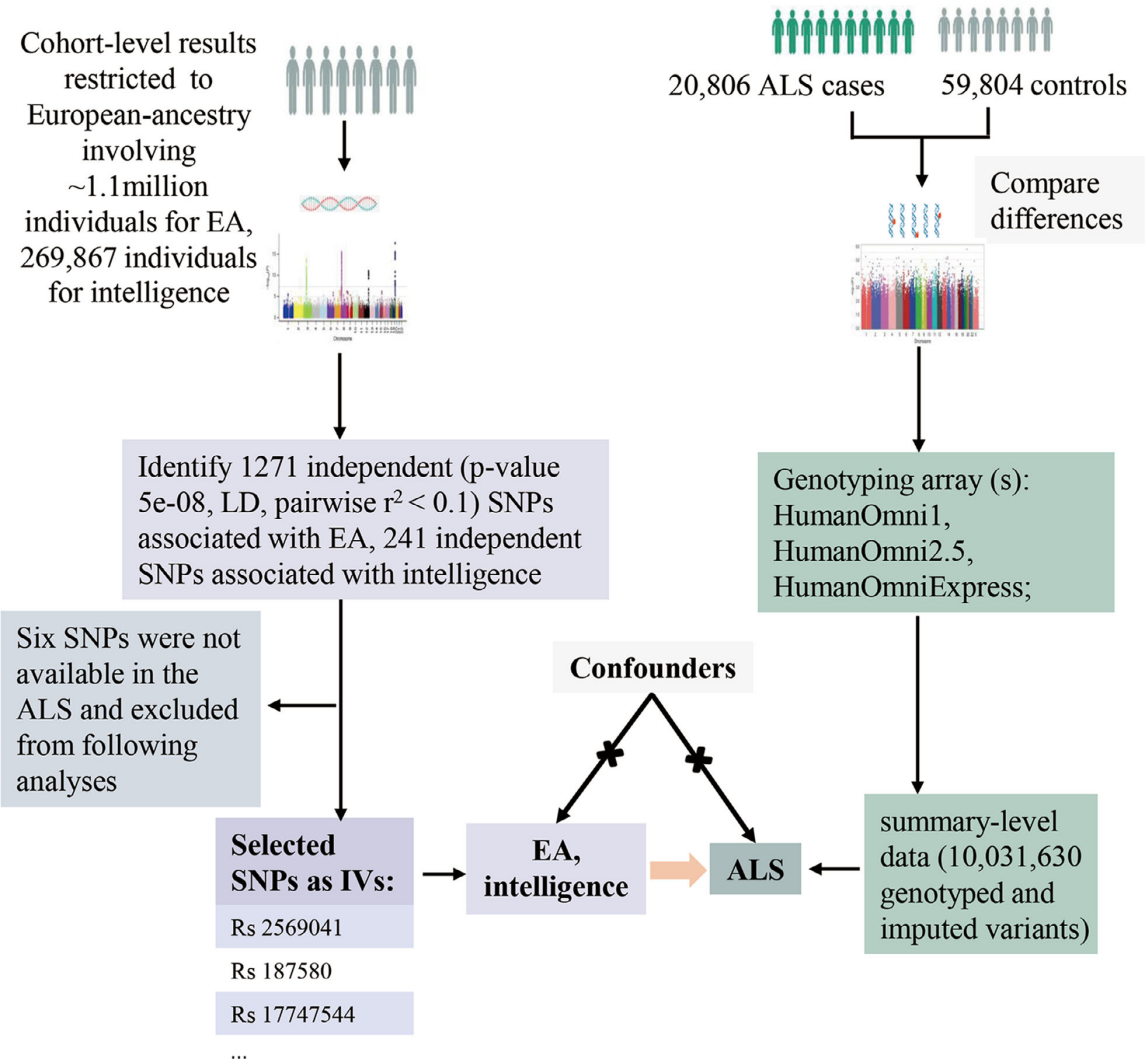
Schematic representation of the study. Assumptions of a two‐sample Mendelian randomization analysis between educational attainment, intelligence and risk of amyotrophic lateral sclerosis. EA: educational attainment; ALS: amyotrophic lateral sclerosis.

A schematic representation of the study is provided in Figure 1. Dependent genetic variants or single nucleotide polymorphisms (SNPs) strongly (*P* < 5 × 10^−8^) and solely associated with EA and intelligence were selected as the instrumental variables (IVs), which applied to the summary‐level results of ALS case–control GWAS.^12^ A unconfounded association between exposures and outcome was expected to be obtained through estimates of genetic variants and outcome.

### Outcome data

The current study is based on publicly available summary statistics data of genetic associations with ALS from the ALS Variant Server (AVS), which is the largest genome‐based GWAS to date, including 20,806 ALS cases and 59,804 controls in populations of European ancestry.^13^ The present MR study was approved by our hospital's Ethical Review Authority.

### Selection of instrumental variables

In total, 1271 independent single SNPs strongly (*P* < 5 × 10^−8^) associated with educational attainment were identified from GWAS of educational attainment involving approximately 1.1 million European individuals.^14^ The effect size of the independent SNPs corresponding to an educational increase was obtained as follows: the median effect size corresponds to 1.7 weeks of schooling per allele and 1.1 and 2.6 weeks at the 5th and 95th percentiles respectively. In total, approximately 11% of the EA variance was explained by the estimated polygenic score derived from these independent SNPs. Furthermore, the genes related to these SNPs are involved in almost all aspects of neuron‐to‐neuron communication.

In addition, 618, 366, and 226 independent lead SNPs related to cognitive phenotypes, including self‐reported math ability (*N* = 564,698), highest math class (*N* = 430,445), and cognitive performance (*N* = 257,841), respectively, were identified.^14^ The highest math class were evaluated by the question “Excluding statistics, what is the most advanced math class you have successfully completed?”; the more difficult the math class taken, the higher the score.

Additionally, 241 independent SNPs strongly (*P* < 5 × 10^−8^) associated with intelligence were obtained from a meta‐analysis of GWAS of intelligence involving 269,867 European individuals.^15^ The ‘independent’ SNPs selection criteria were described as linkage disequilibrium (LD) (pairwise *r* ^2^ < 0.1) in these two studies.^14^, ^15^

In total, nine SNPs related to five traits in ALS were unavailable, and no proxy for SNP in LD (*r* ^2^> 0.9) was identified for these missing SNPs of ALS. These nine SNPs included educational attainment: rs182355396, rs186456786, rs150421637, rs115732722; math ability: rs62079448, rs186538251; highest math class: rs182320519; intelligence: rs1991228, rs13184816 respectively. Thus, this MR analysis of ALS included 1267 SNPs related to educational attainment, 616 SNPs related to math ability, 365 SNPs related to the highest math class, 226 SNPs related to cognitive performance, and 239 SNPs related to intelligence as the IVs.

The selected SNPs were assessed to determine whether they were also significantly associated with key confounders, which may influence the risk of ALS.^8^ The intercept obtained from the MR‐Egger method was evaluated to pleiotropy to ensure that this analysis met the following assumption of two‐sample designs: the IVs were solely linked to ALS via EA and intelligence.^16^, ^17^, ^18^

### Mendelian randomization analysis

The ‘TwoSampleMR’ (version 0.4.25) R package was applied in this study. The full documentation is available online at https://mrcieu.github.io/TwoSampleMR. For each IV described above, an IV ratio estimate was calculated by dividing the effect size estimate of the association between the variant and the risk of ALS by the corresponding estimate of education attainment and intelligence. Then, we applied the inverse–variance weighted method (IVW),^19^ simple median method, weighted median method,^20^ and MR‐Egger regression methods^18^ to these ratio estimates. A Bonferroni corrected significance level computed as 0.05 divided by 5 (i.e., 0.01) was applied.

## Results

**Figure 2:**
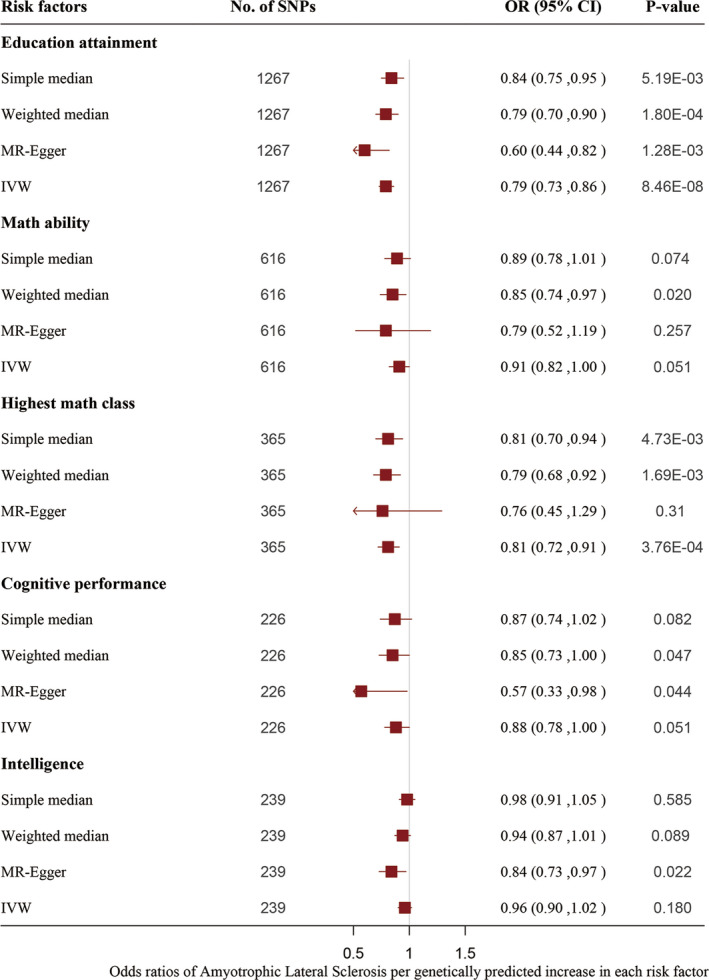
Odds ratio of ALS per genetically predicted increase in educational attainment and intelligence. IVW = inverse‐variance weighted (conventional method). The MR‐Egger method did not detect evidence of directional pleiotropy (*P* = 0.07 for years of education, *P* = 0.79 for highest math class taken).

Genetically predicted higher educational attainment was associated with significant lower odds of ALS. The odds ratio was 0.79 (95% confidence intervals 0.73–0.86; *P* = 8.46e‐08) per year of education completed in the analyses using the IVW method. The associations were consistent in the sensitivity analyses using the simple median and weighted median methods but with less precision; the odds ratio was 0.84 (95% CI: 0.75, 0.95; *P* = 5.19e‐3) and 0.79 (CI: 0.70, 0.90; *P* = 1.80e‐4) per year of education completed. The MR‐Egger analyses showed no evidence of directional pleiotropy (*P* = 0.07) (Figure 2).

The genetically predicted highest math class was associated with a significantly reduced risk of ALS. The odds ratio of ALS was 0.81 (95% CI: 0.72, 0.91; *P* = 3.76e‐4) per 1‐SD increase in the highest math class taken obtained from the IVW regression. In addition, the robustness of the results was confirmed by sensitivity analyses using the simple median and weighted median methods but with less precision as follows: the odds ratio was 0.81 (95% CI: 0.70, 0.94; *P* = 4.73e‐3) and 0.79 (95% CI: 0.68, 0.92; *P* = 1.69e‐3). The intercept obtained from the MR‐Egger analyses showed no evidence of directional pleiotropy (*P* = 0.79).

Math ability, cognitive performance, and intelligence were not associated with the risk of ALS after the Bonferroni correction as follows: the odds ratio and 95% CI were 0.91 (0.82, 1.00; *P* = 0.05), 0.88 (0.78, 1.00; *P* = 0.05), and 0.96 (0.90, 1.02; *P* = 0.18) (Figure 2).

## Discussion

We performed a two‐sample MR analysis to investigate the causal relationship among educational attainment, four cognitive‐related phenotypes, and ALS using summary statistics from large GWASs. This study provides genetic support for a causal association among higher education, highest math class taken, and a reduced risk of ALS. Based on the intercept estimates of the MR‐Egger analysis, there was no evidence of horizontal pleiotropy that likely influenced our results.

Our results are consistent with those reported by previous case–control studies.^1^, ^2^, ^3^, ^4^ However, it is imperative to identify the biological link underlying the association between higher educational attainment and a lower risk of ALS. The SNPs selected in this study were associated with educational attainment or cognitive performance and involved in all aspects of neuron‐to‐neuron communication. In addition, this finding implies that an increased cognitive reserve is more likely to recruit alternative brain networks or cognitive paradigms or use brain structures or networks to offset brain aging.^21^ Similar to the role of the cognitive reserve in Alzheimer's disease, i.e., higher educational attainment is associated with a reduced risk of Alzheimer's disease,^22^ the cognitive reserve was indicated to play a possible role in ALS‐related cognitive impairment.^23^ This association could also be mediated by health behaviors and occupation partially acquired from education; hence, medication use, depression, chronic stress, and potential exposure to occupation hazards could influence the risk of ALS. Considering the ALS‐FTD overlapping genetic spectrum, it is inferred that the predisposition to higher educational attainment may likely to protect individuals from developing FTD, a disease characterized by cognitive and/or behavioral impairment.

Our study is subject to some limitations. First, the subjects involved in the two GWASs were unrelated individuals. It has been found that environmentally mediated parental genetic effects (i.e., passive genotype‐environment correlation) might be a mechanism explaining a considerable proportion of the genome‐wide polygenic score (GPS) prediction in cognitive traits (intelligence and educational achievement) by contrasting within‐ and between‐family GPS prediction.^24^ In addition, the family socio‐economic status is likely a major source of between‐family predictions in genotype‐environment correlations. Thus, genotypes are not inherited randomly at the population level, suggesting that EA exposure in an MR design could lead to an assumption violation because the family environment is associated with the genetic instrument, EA and ALS. Specifically, only a within‐family design can ensure that MR meets its assumptions.^24^ Although genetic data of siblings are not always available, caution should be raised when cognitive performance is assessed to determine whether causal associations with outcomes exist in an MR design. Consistently, the robustness of the results of GWAS of EA was probed by comparing the resulting estimates from within‐family association analyses (22,135 sibling pairs) with those from unrelated individuals (*N* = 1,070,751), suggesting that GWAS estimates of EA may overstate the causal effect sizes.^14^ Second, higher cognitive performance and higher educational attainment were found to share polygenic genetic risk factors with ALS using a LD score regression, indicating a negative correlation between educational attainment and the risk of ALS.^25^ Additionally, Bandres‐Ciga S et al. implemented MR method to survey hundreds of GWAS results (including *Age completed full time education id:UKB‐a:505 and Fluid intelligence score id:UKB‐a:196*) in a large‐scale survey relevant to ALS, and made their results public online https://lng‐nia.shinyapps.io/mrshiny. Neither of these two traits showed significant effects on risk of ALS. Third, although examining the intercept estimates from the MR‐Egger regression also leads to the conclusion that horizontal pleiotropy unlikely influenced the results of the relationship between education and ALS, completely ruling out pleiotropy or an alternative direct causal pathway is a challenge in all MR analyses.^26^ Finally, our study assumed that a linear relationship exists between educational attainment and ALS and did not investigate the nonlinear effects of educational attainment.

## Conflicts of Interest

Nothing to report.
